# Value Placed on Comfort vs Life Prolongation Among Patients Treated With Maintenance Dialysis

**DOI:** 10.1001/jamainternmed.2023.0265

**Published:** 2023-03-27

**Authors:** Susan P. Y. Wong, David K. Prince, Manjula Kurella Tamura, Yoshio N. Hall, Catherine R. Butler, Ruth A. Engelberg, Elizabeth K. Vig, J. Randall Curtis, Ann M. O’Hare

**Affiliations:** 1 Department of Medicine, University of Washington, Seattle; 2 Department of Medicine, Stanford University, Palo Alto, California; 3 Cambia Palliative Care Center of Excellence, Department of Medicine, University of Washington, Seattle

## Abstract

**Importance:**

Patients receiving maintenance dialysis experience intensive patterns of end-of-life care that might not be consistent with their values.

**Objective:**

To evaluate the association of patients’ health care values with engagement in advance care planning and end-of-life care.

**Design, Setting, and Participants:**

Survey study of patients who received maintenance dialysis between 2015 and 2018 at dialysis centers in the greater metropolitan areas of Seattle, Washington, and Nashville, Tennessee, with longitudinal follow-up of decedents. Logistic regression models were used to estimate probabilities. Data analysis was conducted between May and October 2022.

**Exposures:**

A survey question about the value that the participant would place on longevity-focused vs comfort-focused care if they were to become seriously ill.

**Main Outcomes and Measures:**

Self-reported engagement in advance care planning and care received near the end of life through 2020 using linked kidney registry data and Medicare claims.

**Results:**

Of 933 patients (mean [SD] age, 62.6 [14.0] years; 525 male patients [56.3%]; 254 [27.2%] identified as Black) who responded to the question about values and could be linked to registry data (65.2% response rate [933 of 1431 eligible patients]), 452 (48.4%) indicated that they would value comfort-focused care, 179 (19.2%) that they would value longevity-focused care, and 302 (32.4%) that they were unsure about the intensity of care they would value. Many had not completed an advance directive (estimated probability, 47.5% [95% CI, 42.9%-52.1%] of those who would value comfort-focused care vs 28.1% [95% CI, 24.0%-32.3%] of those who would value longevity-focused care or were unsure; *P* < .001), had not discussed hospice (estimated probability, 28.6% [95% CI, 24.6%-32.9%] comfort focused vs 18.2% [95% CI, 14.7%-21.7%] longevity focused or unsure; *P* < .001), or had not discussed stopping dialysis (estimated probability, 33.3% [95% CI, 29.0%-37.7%] comfort focused vs 21.9% [95% CI, 18.2%-25.8%] longevity focused or unsure; *P* < .001). Most respondents wanted to receive cardiopulmonary resuscitation (estimated probability, 78.0% [95% CI, 74.2%-81.7%] comfort focused vs 93.9% [95% CI, 91.4%-96.1%] longevity focused or unsure; *P* < .001) and mechanical ventilation (estimated probability, 52.0% [95% CI, 47.4%-56.6%] comfort focused vs 77.9% [95% CI, 74.0%-81.7%] longevity focused or unsure; *P* < .001). Among decedents, the percentages of participants who received an intensive procedure during the final month of life (estimated probability, 23.5% [95% CI, 16.5%-31.0%] comfort focused vs 26.1% [95% CI, 18.0%-34.5%] longevity focused or unsure; *P* = .64), discontinued dialysis (estimated probability, 38.3% [95% CI, 32.0%-44.8%] comfort focused vs 30.2% [95% CI, 23.0%-37.8%] longevity focused or unsure; *P* = .09), and enrolled in hospice (estimated probability, 32.2% [95% CI, 25.7%-38.7%] comfort focused vs 23.3% [95% CI, 16.4%-30.5%] longevity focused or unsure; *P* = .07) were not statistically different.

**Conclusions and Relevance:**

This survey study found that there appeared to be a disconnect between patients’ expressed values, which were largely comfort focused, and their engagement in advance care planning and end-of-life care, which reflected a focus on longevity. These findings suggest important opportunities to improve the quality of care for patients receiving dialysis.

## Key Points

**Question:**

Are patients’ values around comfort vs life prolongation associated with their engagement in advance care planning and end-of-life care?

**Finding:**

Of the 933 patients receiving maintenance dialysis in this survey study, 452 indicated that they would value comfort-focused care rather than longevity-focused care if they were seriously ill. Differences in these health care values did not translate into substantial differences in either engagement in advance care planning or end-of-life care, both of which suggested a focus on life prolongation.

**Meaning:**

This study found that there appeared to be a disconnect between patients’ expressed values around comfort vs longevity and engagement in advance care planning and end-of-life care.

## Teaser

This survey study evaluate the association of patients’ health care values with engagement in advance care planning and end-of-life care.

## Introduction

Patients treated with maintenance dialysis experience frequent and intensive interactions with the health system, including high rates of hospitalization^1^ and nursing home admission.^2^ Compared with some other groups of seriously ill patients, members of this population spend more time in an intensive care unit and are more likely to receive intensive procedures, such as cardiopulmonary resuscitation (CPR), mechanical ventilation, and artificial enteral nutrition, during the final month of life.^3^ They are also more likely than other populations with serious illness to die in the hospital and less likely to receive hospice care.^3,4^

Existing evidence suggests that the intensive patterns of end-of-life care experienced by patients receiving dialysis may be incongruent with the values, goals, and preferences of individual patients. Prior studies indicate that end-of-life care for patients receiving dialysis are more strongly and consistently associated with system-level and health care professional–level factors than with individual patient characteristics.^3,5,6^ In 1 study, bereaved family members of patients who received dialysis were more likely than those of patients with other serious illnesses to report that their loved one received unwanted care.^4^ Several other studies suggest that patients with advanced kidney disease are more likely to prefer care that is directed at preserving quality of life, relieving pain, and promoting independence rather than care that is focused on increasing longevity.^7,8^ Yet, to our knowledge, little is known about how the value placed on longevity vs comfort shapes how members of this population view and prepare for serious illness or the care they ultimately receive near the end of life. We performed a survey study of patients receiving maintenance dialysis with longitudinal follow-up of decedents to learn about the value they placed on life extension vs comfort and its association with their engagement in advance care planning and the care they went on to receive near the end of life.

## Methods

### Study Population

As described in detail elsewhere,^8^ we conducted a survey study that included questions about a range of different aspects of end-of-life care and level of engagement in advance care planning among patients receiving maintenance dialysis (eAppendix in Supplement 1). In brief, we recruited a pragmatic consecutive sample of adults receiving maintenance dialysis at 31 nonprofit and not-for-profit dialysis units located in the greater metropolitan areas of Seattle, Washington, and Nashville, Tennessee, between 2015 and 2018. In most instances, surveys were administered in person by trained research staff during dialysis sessions, although patients were also given the option to return the completed survey to study staff at a later time. We linked patients’ survey data to their records in the United States Renal Data System (USRDS)—a national comprehensive registry of end-stage kidney disease (ESKD) that includes standardized information on demographic and clinical characteristics submitted by nephrology clinicians around the time of dialysis initiation (CMS-2728 Medical Evidence Form) and after death (CMS-2746 Death Notification Form).^9^ Linked Medicare claims are also available through the USRDS.

**Figure 1.**
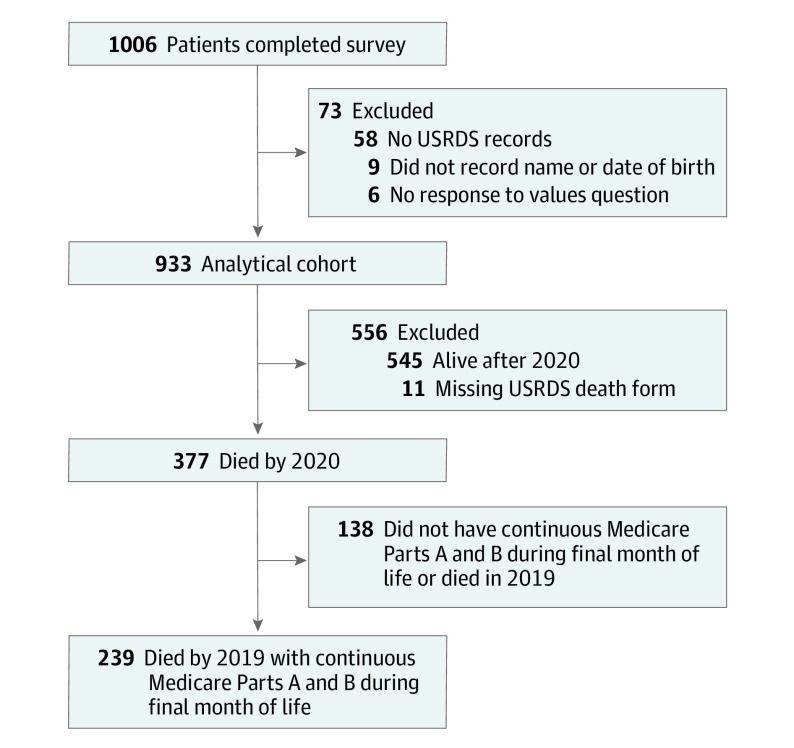
Cohort Derivation USRDS indicates United States Renal Data System.

Patients with limited English proficiency and cognitive impairment were excluded from study participation. A total of 1431 eligible patients were invited to participate, and 1006 completed the survey. For the current analyses (Figure 1), we further excluded 6 patients who did not answer the survey question asking about their values, 9 who did not record their name and/or date of birth on the survey or consent form, and 58 who could not be linked to records in the USRDS, resulting in a final analytical cohort of 933 patients (65.2% of those invited to participate).

The study was approved by the institutional review board at the University of Washington, and patients provided their written informed consent to participate. We followed the Strengthening the Reporting of Observational Studies in Epidemiology (STROBE) reporting guideline.

### Patient Characteristics

Survey responses served as the primary source of information on patients’ age, race and ethnicity (Black, White, or other [included American Indian or Alaska Native, Asian, Native Hawaiian or Other Pacific Islander, and self-specified]), gender, educational background (categorized as high school or less vs some college or more), and self-rated health (categorized as poor or fair vs good, very good, or excellent). Patients were also asked how true the following statement was for them: “my religious or spiritual beliefs are what really lie behind my whole approach to life” (categorized as definitely or tends to be true vs definitely or tends not to be true).

From the USRDS Patients File, we ascertained the duration of dialysis at the time of survey completion based on the date of first ESKD service and supplemented missing survey responses to the question about race (1% of cases). From the USRDS Medical Evidence File, we ascertained the presence of select comorbidities around the time of ESKD onset: congestive heart failure, other heart disease, vascular disease, and type 1 or 2 diabetes.

### Patient Values

The value that patients placed on extending life vs relief from pain and discomfort was based on responses to the following question: “If you were to become very sick in the future and were unable to speak for yourself, would you prefer a plan of medical care that focuses on extending life as much as possible, even if it means having more pain and discomfort, or would you want medical care that focuses on relieving pain and discomfort as much as possible, even if that means not living as long?” Patients also had the option of indicating that they were unsure about which of these options they would prefer. This item was adapted from a survey item developed as part of the SUPPORT (Study to Understand Prognoses and Preferences for Outcomes and Risks of Treatments) trial for seriously ill hospitalized patients.^10,11,12^

### Engagement in Advance Care Planning

Survey participants were asked whether they had ever signed official documents (“e.g. advance directive or living will”) indicating their treatment preferences and surrogate decision-maker if they were to become very sick. They were asked whether they had discussed stopping dialysis and hospice if they were to become sicker or their goals changed. Participants were also asked, if they had to choose at the time of completing the survey, whether they would prefer (categorized as definitely or tends to be true vs definitely not or tends not to be true) to receive CPR or mechanical ventilation, and where they would prefer to die (categorized as hospital, own or relative’s home, or other).

### End-of-Life Care

The time frame for data availability for patients who died during follow-up differed between USRDS files and linked Medicare claims. For all cohort members who died on or before September 30, 2020, we ascertained whether they had discontinued dialysis, died in an inpatient setting, or received hospice care prior to death using the most recently available information in the USRDS Death File. For cohort members who died on or before December 31, 2019, and had continuous Medicare Parts A and B coverage during their final month of life, we additionally ascertained whether they had been hospitalized and whether they had received an intensive procedure (ie, CPR, mechanical ventilation, or artificial enteral nutrition) during the final month of life based on a procedure code search of linked Medicare claims. We used the in-hospital deaths reported in the USRDS Death File to supplement information on hospitalization during the final month of life from Medicare claims.

### Statistical Analysis

For the primary analyses, we compared baseline characteristics, engagement in advance care planning, and patterns of end-of-life care of cohort members who responded that they would value care focused on relieving pain and discomfort (ie, comfort-focused care) vs those who responded that they would value care focused on extending life (ie, longevity-focused care) or were unsure about what kind of care they would value. We estimated probabilities^13^ using logistic regression models.

In sensitivity analyses using multinomial regression models, we repeated these comparisons and estimated probabilities for the 3 groups: patients who responded that they would value comfort-focused care, patients who responded that they would value longevity-focused care, or patients who responded that they were unsure about what kind of care they would value.

Due to sample size limitations, we adopted a parsimonious approach^14^ of adjusting all models for only age, race, and gender. We calculated 95% CIs using quantile-based bootstrapped samples with 10 000 iterations. We used SAS, version 9.4 to construct the analytical data sets and R, version 3.6.2 (R Core Team [2019]; R Project for Statistical Computing) to conduct statistical analyses. Statistical significance was set at 2-sided *P* < .05.

## Results

The mean (SD) age of members of the analytical cohort was 62.6 (14.0) years. Of the 933 patients, 525 (56.3%) were male, and 254 (27.2%) identified as Black. Overall, 452 of 933 cohort members (48.4%) indicated that they would value comfort-focused care, 179 of 933 (19.2%) indicated that they would value longevity-focused care, and 302 of 933 (32.4%) indicated that they were unsure which of these they would prefer.

Compared with participants who would value life prolongation or were unsure about what they would value (Table), those who would value comfort-focused care tended to be older (mean [SD], age, 66 [13] years vs 59 [14] years; *P* < .001) and included a lower proportion who identified as Black (estimated probability, 41.6% [95% CI, 35.8%-47.6%] comfort focused vs 58.4% [95% CI, 52.4%-64.2%] longevity focused; *P* = .002) and a greater proportion with at least some college education or more (estimated probability, 51.5% [95% CI, 47.1%-55.9%] comfort focused vs 48.5% [95% CI, 44.1%-52.9%] longevity focused; *P* = .045) and vascular disease (estimated probability, 54.2% [95% CI, 48.3%-60.0%] comfort focused vs 45.8% [95% CI, 40.0%-51.7%] longevity focused; *P* = .02).

**Table.** Characteristics of Patients

Characteristic	Comfort focused (n = 452)		Longevity focused or unsure (n = 481)		*P* value
	Patients, No. (%)	Estimated probability (95% CI)^a^	Patients, No. (%)	Estimated probability (95% CI)^a^	
Age, mean (SD), y	66 (13)		59 (14)		<.001
Gender					
Female	208 (46.0)	50.6 (45.8-55.4)	200 (41.6)	49.4 (44.6-54.2)	.24
Male	244 (54.0)	46.8 (42.7-51.1)	281 (58.4)	53.2 (48.9-57.3)	
Race					
Black	99 (1.9)	41.6 (35.8-47.6)	155 (32.2)	58.4 (52.4-64.2)	.002
White	298 (65.9)	53.4 (49.1-57.7)	241 (50.1)	46.6 (42.3-50.9)	
Other^b^	55 (12.2)	41.8 (33.7-49.9)	85 (17.7)	58.2 (50.1-66.3)	
Education^c^					
At least some high school or less	191 (42.3)	45.1 (40.5-49.8)	240 (49.9)	54.9 (50.2-59.5)	.045
At least some college or more	260 (57.5)	51.5 (47.1-55.9)	238 (49.5)	48.5 (44.1-52.9)	
Spiritual beliefs shape decisions^c^					
True	323 (71.5)	48.7 (44.9-52.5)	341 (70.9)	51.3 (47.5-55.1)	.70
False	124 (27.4)	47.3 (41.4-53.3)	137 (28.5)	52.7 (46.7-58.6)	
Self-reported health^c^					
Excellent, very good, or good	243 (53.8)	45.8 (41.6-50.1)	287 (59.7)	54.2 (49.9-58.4)	.07
Fair or poor	207 (45.8)	51.7 (46.9-56.4)	194 (40.3)	48.3 (43.6-53.1)	
Congestive heart failure	141 (31.2)	47.9 (41.9-53.8)	135 (28.1)	52.1 (46.2-58.1)	.76
Other heart disease	73 (16.2)	47.0 (39.2-55.0)	68 (14.1)	53.0 (45.0-60.8)	.67
Vascular disease	168 (37.2)	54.2 (48.3-60.0)	119 (24.7)	45.8 (40.0-51.7)	.02
Diabetes mellitus	258 (57.1)	47.6 (43.4-51.8)	274 (57.0)	52.4 (48.2-56.6)	.56
Duration of dialysis, median (IQR), y	2 (1-5)		2 (1-5)		.32

^a^ Based on logistic regression models adjusted for age, race, and gender.

^b^ Included American Indian or Alaska Native, Asian, Native Hawaiian or Other Pacific Islander, and self-specified.

^c^ Percentages were calculated based on a denominator that included missing values for some variables that are not shown.

### Engagement in Advance Care Planning

**Figure 2.**
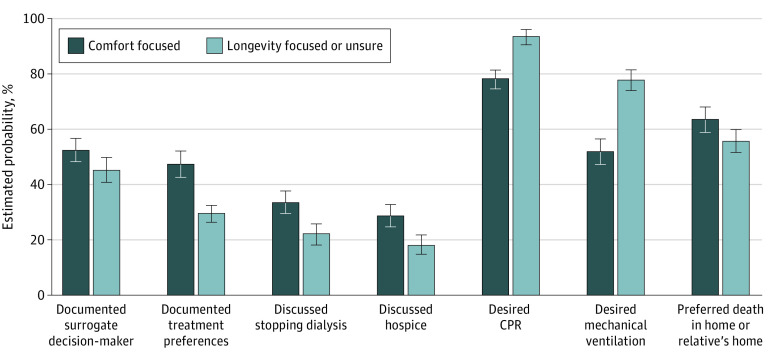
Estimated Probabilities of Advance Care Planning Engagement Among Maintenance Dialysis Patients Error bars indicate 95% CIs. CPR indicates cardiopulmonary resuscitation.

The proportion of participants who indicated that they had documented a surrogate decision-maker was higher for those who would value comfort-focused care than for those who would value longevity-focused care or were unsure (Figure 2) (estimated probability, 52.3% [95% CI, 47.9%-56.8%] comfort focused vs 45.4% [95% CI, 41.0%-50.0%] longevity focused; *P* = .03). Most (578 of 933 [62.0%]) patients indicated that they had not signed documents indicating their treatment preferences, but rates were significantly higher for those who indicated they would value comfort-focused care (estimated probability, 47.5% [95% CI, 42.9%-52.1%] comfort focused vs 28.1% [95% CI, 24.0%-32.3%] longevity focused or unsure; *P* < .001). Most also indicated that they had not discussed stopping dialysis (676 of 933 patients [72.5%]) or hospice (709 of 933 patients [76.0%]), although rates were higher for those who would value comfort-focused care for discussion of dialysis discontinuation (estimated probability, 33.3% [95% CI, 29.0%-37.7%] comfort focused vs 21.9% [95% CI, 18.2%-25.8%] longevity focused or unsure; *P* = .001) and hospice (estimated probability, 28.6% [95% CI, 24.6%-32.9%] comfort focused vs 18.2% [95% CI, 14.7%-21.7%] longevity focused or unsure; *P* < .001). Most patients in both groups indicated that they would want CPR (estimated probability, 78.0% [95% CI, 74.2%-81.7%] comfort focused vs 93.9% [95% CI, 91.4%-96.1%] longevity focused or unsure; *P* < .001) and mechanical ventilation (estimated probability, 52.0% [95% CI, 47.4%-56.6%] comfort focused vs 77.9% [95% CI, 74.0%-81.7%] longevity focused or unsure; *P* < .001), but the proportion was lowest for those who would value comfort-focused care. Regardless of their responses to the question about values, most patients also indicated that they would prefer to die at home or at the home of a relative, but the proportion was higher for those who would value comfort-focused care (estimated probability, 63.5% [95% CI, 59.0%-68.1%] comfort focused vs 55.5% [95% CI, 50.9%-60.0%] longevity focused or unsure; *P* = .02).

### End-of-Life Care

#### All Decedents

During follow-up through September 2020, 377 participants (40.4%) died (a mean [SD] of 1.7 [1.1] years after survey administration) as indicated by a completed USRDS Death Notification Form. Of these 377 participants, 216 (57.3%) had indicated at the time of the survey that they would value comfort-focused care, and 161 (42.7%) that they would value longevity-focused care or were unsure what kind of care they would value.

**Figure 3.**
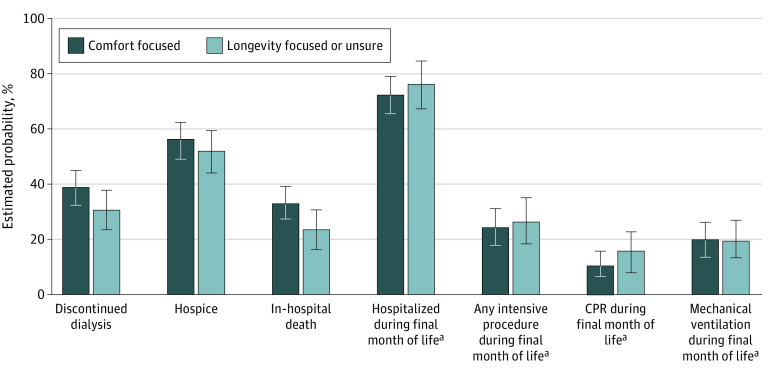
Estimated Probabilities of End-of-Life Care Among Maintenance Dialysis Patients Error bars indicate 95% CIs. CPR indicates cardiopulmonary resuscitation. ^a^Values based on decedents with continuous Medicare Parts A and B during final month of life.

There were no statistically significant differences in the proportion who discontinued dialysis before death (estimated probability, 38.3% [95% CI, 32.0%-44.8%] comfort focused vs 30.2% [95% CI, 23.0%-37.8%] longevity focused or unsure; *P* = .09), received hospice services (estimated probability, 32.2% [95% CI, 25.7%-38.7%] comfort focused vs 23.3% [95% CI, 16.4%-30.5%] longevity focused or unsure; *P* = .07), or died in the hospital setting (estimated probability, 55.7% [95% CI, 49.2%-62.5%] comfort focused vs 52.0% [95% CI, 44.0%-59.8%] longevity focused or unsure; *P* = .48) between patients who would value comfort-focused care and those who would value longevity-focused care or were unsure, although point estimates were higher for those in the former group (Figure 3).

#### Decedents With Medicare Coverage During Final Month of Life

During follow-up through December 2019, 239 participants (25.6%) who died during this interval had continuous Medicare Parts A and B coverage during the final month of life. Of these 239 participants, 136 (56.9%) had indicated that they would value comfort-focused care, and 103 (43.1%) that they would value longevity-focused care or were unsure.

There were no statistically significant differences in rates of hospitalization (estimated probability, 71.8% [95% CI, 64.0%-79.1%] comfort focused vs 76.2% [95% CI, 67.4%-84.1%] longevity focused or unsure; *P* = .45) or receipt of CPR (estimated probability, 9.9% [95% CI, 4.9%-15.3%] comfort focused vs 15.5% [95% CI, 9.2%-22.3%] longevity focused or unsure; *P* = .18), mechanical ventilation (estimated probability, 19.4% [95% CI, 12.9%-26.5%] comfort focused vs 19.0% [95% CI, 11.8%-26.8%] longevity focused or unsure; *P* = .94), or an intensive procedure (estimated probability, 23.5% [95% CI, 16.5%-31.0%] comfort focused vs 26.1% [95% CI, 18.0%-34.5%] longevity focused or unsure; *P* = .64) during the final month of life as a function of how patients had responded to the question about values (Figure 3), although point estimates were generally lower for those who would value comfort-focused care.

### Sensitivity Analyses

Engagement in advance care planning (eTable 1 in Supplement 1) and end-of-life care (eTable 2 in Supplement 1) among participants who were unsure about what kind of care they would value were generally intermediate between the 2 other groups, although more closely approximated those of participants who would value longevity-focused care than participants who would value comfort-focused care.

## Discussion

Among patients receiving maintenance dialysis who responded to a survey question about what kind of care they would value if they were to become seriously ill, the most popular response was for a comfort-focused approach. However, regardless of how study participants responded to the question about their health care values, most had not documented their treatment preferences or participated in other aspects of advance care planning. Further, most participants who died during follow-up received relatively intensive patterns of end-of-life care regardless of their values around comfort vs life prolongation.

Consistent with prior literature on patients with advanced kidney disease,^15,16,17,18,19^ most study participants had not completed an advance directive documenting their treatment preferences or discussed more comfort-oriented approaches to care, such as hospice and stopping dialysis, and most indicated that if they had to decide right now, they would want life-prolonging measures, such as CPR and mechanical ventilation. Advance care planning is a proactive process that aims to clarify patients’ values, goals, and preferences for future medical care to ensure that each individual receives care that is aligned with what is most important to them.^20^ Our findings speak to the obstacles to achieving this ideal and important targets for intervention to improve advance care planning in this population. Other studies have shown that goals-of-care discussions occur infrequently and are often rushed or deferred until precipitated by an illness crisis.^21,22^ The potential benefits and burdens of treatments intended to prolong life are often addressed only superficially and with limited guidance as to how treatments might support patients’ values, if at all.^21,23,24^ Likewise, hospice or forgoing dialysis are discussed infrequently or are presented as options of last resort.^25,26^ Illness trajectories in ESKD can be difficult to predict^27,28^ and discuss with patients.^29^ The quality of these conversations is further hampered by the limited training in effective communication that health care professionals receive.^30^

In addition to highlighting the challenges of eliciting patients’ values and preparing patients for what to expect in terms of their future illness course, our findings speak to the growing debate^31^ about whether advance care planning alone will ensure goal-concordant care in the presence of powerful health system defaults favoring life prolongation.^3,32,33^ Among the patients who died during follow-up, measures of end-of-life care did not differ markedly regardless of how patients had responded to the question about values. No matter whether they had indicated that they would value a longevity-focused or comfort-focused approach, most participants were hospitalized during the final month of life, more than 1 in 5 received at least 1 intensive procedure, and most participants had not stopped dialysis or received hospice care prior to death. Although patients who are uncertain about their values or lack strong care preferences are probably most susceptible to defaults favoring aggressive longevity-focused care,^34^ our findings suggest that even patients who value a comfort-focused approach are not immune to such defaults. Along with earlier studies showing how patient refusal of life-prolonging treatment may be met with resistance from health care professionals,^25,35,36^ our findings raise concern that current defaults toward aggressive care may lead to care that is incongruent with the values held by a substantial number of patients receiving dialysis.

### Limitations

Our findings should be interpreted with the following limitations in mind. First, the present study uses a single question based on a discrete choice model to elicit participants’ values around life prolongation. While this approach can be useful in identifying overriding care values,^37^ the structure of the question does not allow for the possibility that participants might value care directed at extending life and relieving pain and discomfort in equal measure or in different contexts, or hold other values that shape care decisions near the end of life. Additionally, how the survey question inquires patients about their values and how patients responded to this question might not replicate how these discussions unfold between patients and their clinicians. Second, treatments focused on comfort and those focused on longevity are not always mutually exclusive, and some might be used to support both goals.^38^ Third, it is possible that the values that patients expressed at the time of the survey may not be the same as those held near the end of life.^39,40^ Fourth, our findings may not be generalizable to the overall dialysis population because our study was conducted among English-speaking patients receiving mostly in-center hemodialysis from nonprofit and not-for-profit dialysis organizations in 2 metropolitan areas. End-of-life care for patients undergoing dialysis who receive fee-for-service Medicare can also differ from those of patients covered by Medicare Advantage or other forms of insurance.^41^ Fifth, owing to the relatively small number of patients included in our analyses of end-of-life care, our findings on the association between patients’ values and subsequent end-of-life care should be considered exploratory and hypothesis generating. Finally, information about engagement in advance care planning is based on patient self-report, and information about end-of-life care abstracted from the USRDS Death File is reported by the health care professional, both of whom are subject to error and recall bias. Ascertainment of intensive procedures using Medicare claims might also be incomplete.^42^ We also did not confirm whether patients’ expressed preferences for CPR and mechanical ventilation were based on a clear understanding of the risks and benefits of these treatments.

## Conclusions

In conclusion, in this large survey study of patients undergoing maintenance dialysis, most indicated that they would value a comfort-focused rather than longevity-focused approach to care if they were seriously ill. However, differences in how patients reponded to the question about values did not translate into substantial differences in their engagement in advance care planning or the care they received at the end of life. These findings likely reflect the challenges to effective advance care planning and the presence of strong health system defaults favoring longevity-focused over comfort-focused care among members of this population. These findings also suggest important opportunities to better align the care that patients undergoing dialysis receive with their underlying values.

## Notes

Supplement 1. **eAppendix.** Study Survey **eTable 1.** Study Results on Advance Care Planning Behaviors and Treatment Preferences (N=933) **eTable 2.** End-of-Life Care

Supplement 2.
Data Sharing Statement
