# Supplementary material for: Value Placed on Comfort vs Life Prolongation Among Patients Treated With Maintenance Dialysis
Source: JAMA Intern Med. 2023 Mar 27;183(5):462–9. doi: 10.1001/jamainternmed.2023.0265 (PMC10043804; doi:10.1001/jamainternmed.2023.0265)
Supplement: Supplement 1. — eAppendix. Study Survey eTable 1. Study Results on Advance Care Planning Behaviors and Treatment Preferences (N=933) eTable 2. End-of-Life Care [file jamainternmed-e230265-s001.pdf]

Supplemental Online Content

Wong SPY, Prince DK, Kurella Tamura M, et al. Value placed on comfort vs life prolongation among patients treated with maintenance dialysis. *JAMA Intern Med*. Published online March 27, 2023. doi:10.1001/jamainternmed.2023.0265

eAppendix. Study Survey

eTable 1. Study Results on Advance Care Planning Behaviors and Treatment Preferences (N=933)

eTable 2. End-of-Life Care

This supplemental material has been provided by the authors to give readers additional information about their work.

eAppendix. Study survey

Materials include only survey items presented in the current study and do not include all items in the original survey.

© 2023 Wong SPY et al. *JAMA Internal Medicine.*

USRDS Study about Treatment Preferences (UState)
Patient Questionnaire

Thank you for taking part in our study to improve care for patients with kidney disease. We want to hear about the experiences of people receiving dialysis. We hope that by improving our understanding of the experiences of patients on dialysis and their families that we will be able to better help patients receive the type of care they need and want.

This questionnaire covers a number of topics. Some questions are about your health and emotions. Another set of questions are about the care preferences you may have now or if you become very sick. Other questions ask for general information about you, so that we can find out more about the people who are participating in this study. It will take about 30 minutes to complete these questions.

Because many types of people will be answering these questions, some questions may not apply to you. Please feel free to skip any questions that you do not want to answer, or that you feel do not apply to you. Because we have used questions from other surveys, some of the questions may seem quite similar to each other. All of your answers are confidential and will not be shared with anyone else.

Thank you very much for taking the time to complete this survey.

Personal information

Your name: ______________ ______________ __________________

First Middle Family name

Your date of birth: ____/_____/_____

Mo. / Day / Year

Your social security number: ______ ____ ______ *(only include if you agree that your social security number can be used for linkage to USRDS)*

© 2023 Wong SPY et al. *JAMA Internal Medicine.*

Section A: Your Overall Health and Symptoms

This section asks about your overall health and symptoms that you have had in the last week.

A1. In general, would you say your health is: ***(Check only one answer)***

□ Excellent
□ Very good
□ Good
□ Fair
□ Poor

Section C: Planning for Serious Illness

This section asks about planning for your future healthcare if you were to become very sick in the future.

C1. Do you have a person who could make medical decisions for you if you were to become very sick and were unable to speak for yourself? *(This is known as a surrogate decision-maker, durable power of attorney, or DPOA)* ***(Check only one answer)***

□ I have not thought about this
□ I have thought about this, but *have not decided who* this would be
□ I know who this would be, but *have not asked* him/her
□ I have asked someone, but *have not signed official papers* naming him/her as the person who will make medical decisions for me
□ I have signed official papers naming someone to make medical decisions for me (e.g., as part of a living will or advance directive), but *have not discussed* this with him/her
□ I have signed official papers naming someone to make medical decisions for me (e.g., as part of a living will or advance directive), and *have discussed* this with him/her

C2. Have you thought about the kinds of treatments that you would want or not want if you were to become very sick and were unable to speak for yourself? ***(Check all that apply)***

□ I have not thought about this
□ I have thought about this, but *have not talked to anyone* about it
□ I have talked about this with a friend or family member, but *have not signed official papers*
□ I have talked about this with a doctor or other healthcare provider, but *have not signed official papers*
□ I have signed official papers documenting my preferences (e.g., living will or advance directive), but *have not talked with any friends or family members* about this
□ I have signed official papers documenting my preferences (e.g., living will or advance directive), *and have talked with at least one friend or family member* about this

© 2023 Wong SPY et al. *JAMA Internal Medicine.*

C3. If you were to become very sick in the future and were unable to speak for yourself, would you prefer a plan of medical care that focuses on extending life as much as possible, even if it means having more pain and discomfort, or would you want a plan of medical care that focuses on relieving pain and discomfort as much as possible, even if that means not living as long? ***(Check only one answer)***

□ Extending life, even if that means having more pain and discomfort
□ Relieving pain and discomfort as much as possible, even if that means not living as long
□ I'm not sure which I would choose

C4. If you had to decide right now, would you want CPR (cardiopulmonary resuscitation) if your heart were to stop beating? ***(Check only one answer)***

□ Definitely yes
□ Probably yes
□ Probably not
□ Definitely not

C5. If you had to decide right now, would you want to be placed on a breathing machine (ventilator or respirator) if you became so sick that you could not breathe on your own? ***(Check only one answer)***

□ Definitely yes
□ Probably yes
□ Probably not
□ Definitely not

C6. If you had to decide right now, where would you prefer to die if circumstances allowed you to choose? ***(Check only one answer)***
□In my own home
□In the home of a relative or friend
□In a hospital
□In a nursing home
□Other: ________________________________ (describe)

C9. Have you <u>*ever had a discussion*</u> about the option of stopping dialysis if you were to become sicker, or if your goals changed? ***(Check all answers that apply)***
□ Yes, with my kidney doctor
□ Yes, with my primary care doctor
□ Yes, with a nurse
□ Yes, with a social worker
□ Yes, with another healthcare provider: _______________(describe)
□ Yes, with a friend or family member
□ No, I have never had a discussion about this with anyone

C11. Have you <u>*ever had a discussion*</u> about the option of receiving hospice care if you were to become sicker or if your goals changed? (This is care that is focused on trying to keep people comfortable toward the end of life rather than trying to prolong life.) ***(Check all answers that apply)***

□ Yes, with my kidney doctor
□ Yes, with my primary care doctor
□ Yes, with a nurse
□ Yes, with a social worker
□ Yes, with another healthcare provider: ________________(describe)
□ Yes, with a friend or family member
□ No, I have never had a discussion about this with anyone

© 2023 Wong SPY et al. *JAMA Internal Medicine.*

Section D: About You

The next questions provide us with information about you so that we will be able to describe the people who completed this questionnaire.

D1. What is your gender?

□ Female
□ Male
□ Other: ________________________________ (describe)

D2. What ethnicity do you consider yourself? ***(Check only one answer)***

□ Non-Hispanic
□ Hispanic

D3. What race do you consider yourself? ***(Check only one answer)***

□ White
□ Black or African American
□ Asian
□ American Indian or Alaskan native
□ Native Hawaiian or other Pacific Islander
□ Other: ________________________________ (describe)

D4. What is the highest level of education you have completed? ***(Check only one answer)***

□ 8th grade or less
□ Some high school
□ Graduated from high school
□ Graduated from college, community college or trade school
□ Other: ________________________________ (describe)

D5. How true is the following statement for you? "My religious or spiritual beliefs are what really lie behind my whole approach to life." ***(Check only one answer)***

□ Definitely true
□ Tends to be true
□ Tends not to be true
□ Definitely not true

© 2023 Wong SPY et al. *JAMA Internal Medicine.*

eTable 1. Survey results on advance care planning behaviors and treatment preferences (N=933)

	Relief from pain and discomfort n=452		Extending life n=179		Unsure n=302		
	N (%)	Predicted probability (95% CI)	N (%)	Predicted probability (95% CI)	N (%)	Predicted probability (95% CI)	p-value[a]
Durable power of attorney	246 (54.4)	52.2 (47.7, 56.7)	67 (37.4)	47.9 (40.5, 55.4)	121 (40.1)	44.0 (38.3, 49.7)	0.07
Advance directive	228 (50.4)	47.4 (42.8, 51.9)	48 (26.8)	32.4 (25.3, 39.7)	75 (24.8)	25.8 (20.9, 30.8)	<0.001
Discussed stopping dialysis	155 (34.3)	33.3 (29.1, 37.7)	38 (21.2)	22.6 (16.2, 29.1)	64 (21.2)	21.5 (17.0, 26.2)	0.001
Discussed hospice	134 (29.6)	28.6 (24.6, 32.8)	31 (17.3)	18.5 (13.0, 24.6)	53 (17.5)	18.0 (13.7, 22.4)	<0.001
Definitely or probably prefer CPR	338 (74.8)	78.0 (74.2, 81.6)	175 (97.8)	97.6 (94.5, 100.0)	277 (91.7)	92.2 (89.0, 95.2)	<0.001
Definitely or probably prefer mechanical ventilation	220 (48.7)	51.8 (47.2, 56.5)	162 (90.5)	88.6 (83.3, 93.4)	216 (71.5)	72.3 (67.2, 77.2)	<0.001
Preferred death in own or relative's home	292 (64.6)	63.5 (58.9, 68.0)	96 (53.6)	57.3 (49.8, 64.6)	156 (51.7)	54.5 (48.7, 60.2)	0.05

[a] Based on multinomial logistic regression models adjusted for age, race, and gender
Values are adjusted predicted probabilities and their 95% confidence intervals unless where otherwise indicated
Abbreviations: IQR, interquartile range; CI, confidence interval; CPR, cardiopulmonary resuscitation

© 2023 Wong SPY et al. *JAMA Internal Medicine.*

eTable 2. End-of-life care

	Relief from pain and discomfort		Extending life		Unsure		
	n (%)	Predicted probability (95% CI)	n (%)	Predicted probability (95% CI)	n (%)	Predicted probability (95% CI)	p-value[a]
Died by 2020 (N=377)	216 (57.3)		62 (16.4)		99 (26.3)		
Discontinued dialysis	88 (40.7)	38.4 (32.0, 44.9)	13 (21.0)	26.1 (14.3, 39.1)	31 (31.3)	32.4 (23.6, 41.5)	0.01
In-hospital death	118 (54.6)	55.7 (49.0, 62.2)	33 (53.2)	49.6 (36.4, 62.7)	53 (53.5)	53.3 (43.5, 63.2)	0.71
Hospice	66 (30.6)	32.2 (26.0, 38.6)	--[b]	--	20 (20.2)	22.7 (14.8, 31.2)	0.16
Died by 2019 with continuous Medicare A & B during final month of life (N=239)	136 (56.9)		34 (14.2)		69 (28.9)		
Hospitalized during final month of life	168 (77.8)	71.7 (63.9, 79.1)	54 (87.1)	82.0 (68.1, 94.0)	76 (76.8)	73.4 (62.3, 83.9)	0.46
Any intensive procedure during final month of life	30 (22.1)	23.5 (16.7, 31.1)	--[b]	--	19 (27.5)	26.2 (16.5, 36.7)	0.90
CPR during final month of life	12 (8.8)	9.9 (5.0, 15.4)	--[b]	--	13 (18.8)	16.7 (9.0, 25.8)	0.36
Mechanical ventilation during final month of life	25 (18.4)	19.4 (12.8, 26.4)	--[b]	--	14 (20.3)	19.2 (10.4, 28.8)	1.00

[a] Based on multinomial logistic regression models adjusted for age, race, and gender
[b] Cell counts (n<11) insufficient to support analyses
Values are adjusted predicted probabilities and their 95% confidence intervals unless where otherwise indicated
Abbreviations: IQR, interquartile range; CI, confidence interval; CPR, cardiopulmonary resuscitation

© 2023 Wong SPY et al. *JAMA Internal Medicine.*
