# Supplementary material for: Value Placed on Comfort vs Life Prolongation Among Patients Treated With Maintenance Dialysis
Source: JAMA Intern Med. 2023 Mar 27;183(5):462–9. doi: 10.1001/jamainternmed.2023.0265 (PMC10043804; doi:10.1001/jamainternmed.2023.0265)
Supplement: Supplement 2. — Data Sharing Statement [file jamainternmed-e230265-s002.pdf]

Data Sharing Statement

Wong. Value Placed on Comfort vs Life Prolongation Among Patients Treated With Maintenance Dialysis. *JAMA Intern Med*. Published March 27, 2023.
doi:10.1001/jamainternmed.2023.0265

Data
Data available: No
